# Crystal structure of dihydrofolate reductase from the emerging pathogenic fungus *Candida auris*

**DOI:** 10.1107/S2059798323004709

**Published:** 2023-07-10

**Authors:** Tim Kirkman, Alice Sketcher, Vinicius de Morais Barroso, Kelly Ishida, Manuela Tosin, Marcio Vinicius Bertacine Dias

**Affiliations:** a Department of Chemistry, University of Warwick, Coventry CV4 7AL, United Kingdom; b Department of Microbiology, Institute of Biomedical Science, University of Sao Paulo, Avenida Professor Lineu Prestes 1374, São Paulo-SP 05508-000, Brazil; Osaka University, Japan

**Keywords:** *Candida auris*, dihydrofolate reductase, crystallography, antifolates

## Abstract

*Candida auris* has emerged as a global health problem with a dramatic spread by nosocomial transmission and a high mortality rate. Antifungal therapy for *C. auris* infections is currently limited due to widespread resistance to fluconazole and amphotericin B and increasing resistance to the front-line drug echinocandin. Therefore, new treatments are urgently required to combat this pathogen. Dihydrofolate reductase (DHFR) has been validated as a potential drug target for *Candida* species, although no structure of the *C. auris* enzyme (CauDHFR) has been reported. Here, crystal structures of CauDHFR are reported as an apoenzyme, as a holoenzyme and in two ternary complexes with pyrimethamine and cycloguanil, which are common antifolates, at near-atomic resolution. Preliminary biochemical and biophysical assays and antifungal susceptibility testing with a variety of classical antifolates were also performed, highlighting the enzyme-inhibition rates and the inhibition of yeast growth. These structural and functional data might provide the basis for a novel drug-discovery campaign against this global threat.

## Abstract 2

The first structures of *Candida auris* dihydrofolate reductase at near-atomic resolution are described, including apo and holo forms and complexes with two antifolate drugs: pyrimethamine and cycloguanil. *C. auris* is a highly significant globally emerging and resistant fungal pathogen and there is a great demand for research and progress towards novel therapeutics.

## 1. Introduction

*Candida auris* is an emergent fungal pathogen that was first described in Japan in 2009; since then it has been detected across the world, although its true prevalence and the associated mortality rate remain unclear as current methods of identification struggle to differentiate it from other non-*C. albicans* species (Du *et al.*, 2020 ▸).

*C. auris* is considered to be a significant global threat due to its high resistance to antifungals and its great ability for intra-hospital and inter-hospital transmission, which arises from its high capacity for adhesion and its tolerance of disinfectants (Černáková *et al.*, 2021 ▸; Watkins *et al.*, 2022 ▸). Moreover, *C. auris* isolates already show 99% and 63% resistance to the commonly used antifungals fluconazole and amphotericin B, respectively. The main mechanisms for azole resistance appear to arise from efflux-pump overexpression, either of ATP-binding cassettes (ABCs) or of major facilitator superfamily (MFS) transporters, or even from the point mutation or overexpression of ERG11, a primary target of azole inhibition that is involved in ergosterol biosynthesis (Sharma *et al.*, 2016 ▸; Rybak *et al.*, 2019 ▸; Healey *et al.*, 2018 ▸). The resistance mechanisms towards polyenes are less well understood, although mutations in the *ERG2*, *ERG3*, *ERG5*, *ERG6* and *ERG11* genes have been associated with a reduction in polyene efficacy (Rhodes *et al.*, 2018 ▸). Currently, echinocandins are recommended as the first-line treatment for invasive candidiasis by *C. auris*, but resistance to this class of antifungals is also rapidly emerging (Fernandes *et al.*, 2022 ▸). The emergence of pan-resistant strains and difficulty in controlling *C. auris* had led to an urgent need for alternative therapeutic options (Fakhim *et al.*, 2018 ▸). In this regard, due to its potential to cause nosocomial outbreaks and lead to mortality, *C. auris* is considered to be a fungal pathogen of critical priority by the World Health Organization, emphasizing the need for research to overcome antifungal resistance and mistakes in its diagnosis (World Health Organization, 2022 ▸).

Dihydrofolate reductase (DHFR) is a key and essential enzyme in the folate pathway that converts dihydrofolate (DHF) to tetrahydrofolate (THF) (Supplementary Fig. S8; Raimondi *et al.*, 2019 ▸). This coenzyme is required for a variety of cellular processes, including the synthesis of purine involved in nucleic acid and amino-acid biosynthesis. DHFR inhibitors are already used to treat a wide variety of diseases, including antimalarial (trimethoprim, TMP), anticancer (methotrexate, MTX) and antifungal treatments, such as pyrimethamine (PYR) in the treatment of *Pneumocystis jirovecci* pneumonia (Schürmann *et al.*, 2001 ▸). Recently, DHFR was validated as a target for antimicrobial development in several *Candida* species, including *C. albicans* and *C. glabrata* (DeJarnette *et al.*, 2020 ▸). In *C. albicans*, MTX, a DHF analog commonly used to treat several cancers and immune diseases, was shown to inhibit *C. albicans* DHFR with an IC_50_ of 1 n*M* *in vitro*, but difficulty in cell permeability has restricted it from becoming a treatment option. Another clinically relevant antifolate, PYR, has been shown to have a minimum inhibitory concentration (MIC) of 64 µg ml^−1^ against *C. albicans*, with a further reduction in MIC when used in combination with azoles (Navarro-Martínez *et al.*, 2006 ▸).

The development of a series of propargyl-based inhibitors of *C. glabrata* identified two compounds with subnanomolar inhibitory activity against DHFR. These compounds have strong antifungal activities of 4 and 8 µ*M*, while retaining low mammalian cell toxicity due to the exploitation of a unique hydrophobic pocket near the active site, which was discovered based on analysis of crystal structures of *C. glabata* DHFR (Liu *et al.*, 2008 ▸). DHFRs from both *C. glabata* (CglDHFR) and *C. albicans* (CalDHFR) have been demonstrated to be essential for the survival of these fungi. This strongly indicates that this target should also be crucial for the viability of *C. auris* based on the similarity of the metabolic profiles of these three species. Thus, studies aiming to identify novel compounds targeting *C. auris* DHFR (CauDHFR) might be a promising strategy to combat this difficult-to-treat infection.

In this study, we performed a structural and functional analysis of CauDHFR in the presence of classical antifolates, in particular pyrimethamine (PYR), trimethoprim (TMP) and cycloguanil (CYG). Overall, we observed that these compounds are able to bind to CauDHFR with moderate affinities and also have an MIC of as low as 125 µg ml^−1^ in *C. auris*. We determined structures of CauDHFR in the apo and holo forms and in complex with PYR and CYG at high resolution, which could be a starting point for the structural and rational design of new molecules with potential for use in the treatment of *C. auris* infections.

## 2. Materials and methods

### 2.1. Expression and purification of CauDHFR

A codon-optimized transcription region from the *folA* gene that encodes DHFR from *C. auris* subcloned into a pET-28a vector was obtained from Twist Bioscience (pET-28a-CauDHFR). BL21(DE3) competent *Escherichia coli* cells were transformed with pET-28a-CauDHFR. The transformed cells were grown in lysogeny broth (LB) medium supplemented with 50 µg ml^−1^ kanamycin at 37°C until the OD_600_ reached 0.5–0.7. Protein expression was induced by adding 0.2 m*M* isopropyl β-d-1-thiogalactopyranoside (IPTG) for 18 h at 18°C. The cells were spun down at 2000*g* for 20 min and the cell pellet was resuspended in HEPES buffer (50 m*M* HEPES, 100 m*M* NaCl pH 7.5) and homogenized. The cells were then lysed and subsequently centrifuged at 27 000*g* for 15 min. The supernatant was collected and filtered through a 0.22 µm filter. The cell lysate was loaded onto a column packed with HisPur Ni–NTA Agarose beads and washed with two column volumes of buffer *A* (50 m*M* HEPES, 100 m*M* NaCl, 5 m*M* imidazole pH 7.5). The protein was eluted with a gradient of buffer *B* (50 m*M* HEPES, 100 m*M* NaCl, 500 m*M* imidazole pH 7.5). Fractions containing CauDHFR were collected, concentrated and loaded onto a HiPrep 26/60 S200 Superdex column. The column was washed with HEPES buffer and protein elution was monitored with an ÄKTA UV spectrophotometer at 280 nm. Fractions deemed to contain CauDHFR were pooled, concentrated to 20 mg ml^−1^, flash-frozen in liquid nitrogen and stored at −80°C.

### 2.2. Thrombin cleavage

10 mg purified CauDHFR was buffer-exchanged into cleavage buffer (50 m*M* Tris–HCl pH 8.0, 10 m*M* CaCl_2_). 200 µl of a 50%(*v*/*v*) suspension of thrombin–agarose resin (Merck) was washed with 500 µl cleavage buffer three times. The resin was resuspended in 200 µl 10× cleavage buffer and CauDHFR was added. The final volume was increased to 2 ml using water. The mixture was incubated at room temperature with gentle agitation for 18 h. The mixture was centrifuged for 5 min at 500*g*, after which the supernatant containing the cleaved protein was removed. The protein was then loaded onto a HiPrep 26/60 S200 Superdex column. The column was washed with HEPES buffer (50 m*M* HEPES, 100 m*M* NaCl pH 7.5) and protein elution was monitored with an ÄKTA UV spectrophotometer at 280 nm. Fractions deemed to contain CauDHFR were pooled, concentrated to 15 mg ml^−1^, flash-frozen in liquid nitrogen and stored at −80°C. Protein mass spectrometry for the hexahistine-tagged protein gave the following results: expected, 25 894.63 Da; found, 25 761.94 Da (−methionine) and 25 940.94 Da (−methionine, +glucono­ylation). Thrombin-cleaved protein: expected, 24 012.58 Da; found, 24 012.18 and 24 027.18 Da.

### 2.3. Crystallization trials

All crystallization trials were performed by the hanging-drop vapour-diffusion method using an Xtal3 Mosquito robot and a variety of crystallization kits, in particular PACT and JCSG-*plus*. The experiments were performed in MRC 96-well plates, adding 50 µl of each precipitant to each of the wells of the plate. A 600 nl drop was set up using equal volumes (300 nl) of well precipitant and protein solution at 10 mg ml^−1^. Optimization was performed in Linbro plates, and the best conditions involved the use of CauDHFR apoenzyme with a noncleaved His tag at 10 mg ml^−1^ mixed with 2 m*M* NADPH in HEPES buffer (50 m*M* HEPES, 100 m*M* NaCl pH 7.5). The precipitant mixture consisted of 1.6 *M* sodium citrate pH 6.3. Protein:precipitant ratios of 1:2, 1:1 and 2:1 were used. No cryogenic solution was added to the crystals. For the co-crystal with NADPH, the His-tagged protein mixture consisted of 10 mg ml^−1^ CauDHFR and 2 m*M* NADPH in HEPES buffer. The precipitant mixture consisted of 0.2 *M* sodium nitrate, 20% PEG 3350. Protein:precipitant ratios of 1:2, 1:1 and 2:1 were used and 20% ethylene glycol was used to obtain a cryogenic solution. Finally, for the co-crystal with NADPH and PYR, a His-tag-free protein mixture consisting of 10 mg ml^−1^ CauDHFR and 2 m*M* NADPH in HEPES buffer was used. The precipitant mixture consisted of 0.2 *M* sodium nitrate, 20% PEG 3350. Protein:precipitant ratios of 1:2, 1:1 and 2:1 were used and 20% ethylene glycol was used to obtain a cryogenic solution.

### 2.4. Data collection, processing, structure determination and analysis

X-ray data collection was performed at Diamond Light Source, UK. The data were processed by *XDS* and scaled using *AIMLESS* from the *CCP*4 suite (Kabsch, 2010 ▸; Evans & Murshudov, 2013 ▸; Agirre *et al.*, 2023 ▸). The structures were solved by molecular replacement using *Phaser* from the *Phenix* suite (McCoy, 2007 ▸; Liebschner *et al.*, 2019 ▸) with PDB entry 3qlw as a search model (Paulsen *et al.*, 2011 ▸). Crystallographic refinement was performed using *phenix.refine* followed by manual inspection using *Coot* (Emsley *et al.*, 2010 ▸; Afonine *et al.*, 2018 ▸). The quality of the models was checked by *MolProbity* (Williams *et al.*, 2018 ▸). Figures were prepared using *PyMOL* (version 1.8; Schrödinger).

### 2.5. Enzyme-inhibition assay (NADPH-consumption assay)

The activity of CauDHFR was measured in a 300 µl assay consisting of 50 m*M* HEPES, 100 m*M* NaCl, 5 m*M* tris(2-carboxyethyl)phosphine (TCEP), 1 mg ml^−1^ bovine serum albumin (BSA) at pH 7.5 with various concentrations of NADPH and DHF in the range 0–100 µ*M*. A final concentration of 50 m*M* CauDHFR was used in the assay. The assay was started by the addition of DHF, and NADPH oxidation was monitored at 340 nm using a Hidex Sense microplate reader. All measurements were performed at room temperature and in triplicate. Inhibitors were dissolved in 100% DMSO and added to the mixture 5 min prior to the addition of DHF.

### 2.6. Differential scanning fluorimetry (DSF)

DSF assays were performed using an AriaMx Real-Time PCR System. 96-well plates were used; each well contained a total of 30 µl consisting of 95%(*v*/*v*) HEPES buffer, 5%(*v*/*v*) DMSO, 5.0× SYPRO Orange dye and 1 m*M* NADPH. The final concentration of CauDHFR in each well was 30 µ*M*. The final inhibitor concentration in each well was 5 m*M*. Three wells in each run were used as a negative control, with no inhibitor added (but retaining 5% DMSO). For assays, the plate was heated from 25 to 90°C in 0.2°C increments every 24 s. The fluorescence intensity of SYPRO Orange dye was monitored, with excitation and emission wavelengths of 490 and 575 nm, respectively, as a function of temperature. Δ*T*_m_ was calculated from the difference between the average value of all negative controls and that of the protein in the presence of compounds.

### 2.7. Determination of the minimum inhibitory concentration (MIC) 

The antifungal activity of DHFR inhibitors was evaluated against *C. albicans* (SC 5314), *C. glabrata* (ATCC 2001) and *C. auris* (CBS 12766) by the broth microdilution technique (CLSI, 2017 ▸). DHFR inhibitors were serially diluted (1:2) in RPMI 1640 medium buffered with 0.165 *M* 3-(*N*-morpho­lino)propanesulfonic acid in 96-well flat-bottom plates and yeast suspension was added to the wells to obtain a final yeast concentration of 0.5–2.5 × 10^3^ CFU ml^−1^ and final concentrations of DHFR inhibitors in the range 1–500 µg ml^−1^. The plates were incubated at 35°C for 24 h to visually determine the minimum inhibitory concentration (MIC), which was defined as the lowest concentration that inhibits 50% of fungal growth.

## 3. Results and discussion

### 3.1. Overall structures of *C. auris* DHFR

Crystals of different complexes of CauDHFR have been obtained and they diffracted to resolutions of between 1.3 and 2.4 Å in different space groups (Table 1 ▸). Crystals of apo and holo CauDHFR have a single protomer in the asymmetric unit, while the ternary complexes CauDHFR–NADPH–PYR and CauDHFR–NADPH–CYG contained two protein molecules which are independent and do not form a quaternary structure, according to the *PISA* server (Krissinel & Henrick, 2007 ▸). The structure of apo CauDHFR was determined by molecular replacement using the structure of CalDHFR as a model, while the structures of the CauDHFR–NADPH, CauDHFR–NADPH–PYR and CauDHFR–NADPH–CYG complexes were also solved by molecular replacement using the structure of apo CauDHFR as a model. Further processing and refinement statistics are given in Table 1 ▸. In order to obtain the structures of the CauDHFR–NADPH–PYR and CauDHFR–NADPH–CYG ternary complexes, the N-terminal His tag of the protein needed to be removed since analysis of the crystal packing of the CauDHFR–NADPH complex showed that the N-terminus of the adjacent symmetric protomer extends into the substrate-binding site, blocking ligand access.

**Figure 1:**
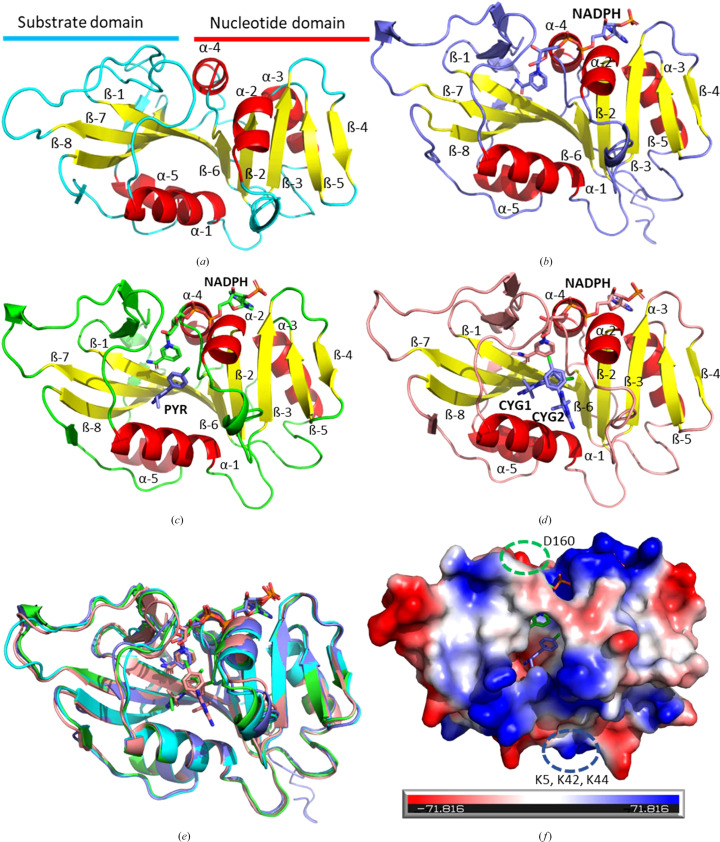
Overall structure of CauDHFR. (*a*) CauDHFR apoenzyme structure (cyan), with the subdomains highlighted in red and blue (α-helices shown in red and β-sheets shown in yellow; PDB entry 7zzx). (*b*) CauDHFR holoenzyme structure (blue; α-helices shown in red and β-sheets shown in yellow; PDB entry 8a0n). (*c*) Crystal structure of CauDHFR in complex with NADPH and PYR (green; PYR is in blue; α-helices are shown in red and β-­sheets in yellow; PDB entry 8a0z). (*d*) Crystal structure of CauDHFR in complex with NAPDH and CYG (salmon; CYG is in blue; α-helices are shown in red and β-sheets in yellow; PDB entry 8crh). (*e*) Superposition of PDB entries 7zzx (cyan), 8a0n (blue) and 8a0z (green). (*f*) Electrostatic surface of PDB entry 8a0z.

The overall tertiary structures of the four structures reported here are very similar (Figs. 1 ▸ *a*–1 ▸ *e*). Overall, CauDHFR has a Rossmann fold constituted by a central eight-stranded β-sheet flanked by five α-helices (Fig. 1 ▸). This is also a similar to the tertiary structure observed for CglDHFR and CalDHFR (r.m.s.d.s of 0.65 and 0.50 Å, respectively). In CauDHFR, α-helices 1–5 are constituted by residues Lys30–Ser41, Arg55–Leu60, Phe95–Asp105, Ser123–Leu131 and His171–His177, respectively, while β-sheets 1–8 include residues Lys5–Leu13, Asn49–Gly54, Leu72–Ser77, Asp82–Asp85, Gly88–Phe92, Lys116–Ile119, Asn137–Phe145 and Tyr192–Lys201, respectively (Fig. 1 ▸).

Analysis of the electrostatic surface of CauDHFR shows that the protein is predominantly positively charged, particularly at the DHF binding site. This would be expected because of the negative charge of the glutamate moieties of the substrate that binds near the exterior of the enzyme. Other regions of the protein have a predominance of negative charge, which is also complementary to the positive charge of the pteridine ring of DHF (Fig. 1 ▸ *f*).

The active site is located in a cleft formed by α-helix 1, α-helix 2, α-helix 4 and β-sheet 1, similar to CglDHFR and CalDHFR. As classified in *E. coli* DHFR, CauDHFR could also be designated to have two subdomains: the nucleotide subdomain, in which the NADPH sits, and the substrate-binding subdomain, which is predominantly formed by α-helix 1, α-helix 2 and β-sheet 1 (Fig. 1 ▸ *a*).

Superposition of the four different CauDHFR structures shows that ligands do not promote large conformation changes in this enzyme. Superposing the structures described here, the ternary PYR complex with the apoenzyme, the holo­enzyme and the ternary CYG complex, gives r.m.s.d.s. of about 0.63, 0.61 and 0.41 Å, respectively. This is different from human and *Mycobacterium tuberculosis* DHFRs (Fig. 1 ▸ *e*), in which large conformational changes are observed (Dias *et al.*, 2014 ▸; Tuttle *et al.*, 2014 ▸). Thus, CauDHFR should adopt a more rigid structure as in the DHFRs from *E. coli* and *Staphylococcus aureus* (Behiry *et al.*, 2014 ▸; Shrimpton & Allemann, 2002 ▸). Similarly to the observations for *E. coli* DHFR, only loop movements are observed in the different CauDHFR structures reported here. In *E. coli* DHFR the major differences are observed between residues 15 and 25, a region that is adjacent to the NADPH binding site and the DHF binding site, in which a sizable backbone movement between the open, closed and occluded conformations occurs (Supplementary Fig. S1). CauDHFR is less flexible in this region but has slight conformational shifts in other loops, particularly those between α-helix 1 and β-sheet 2, between β-sheet 4 and β-sheet 5, and between β-sheet 7 and α-helix 5 (Fig. 1 ▸ *e*).

### 3.2. Comparison of the CauDHFR ternary complex with DHFRs from other *Candida* species

**Figure 2:**
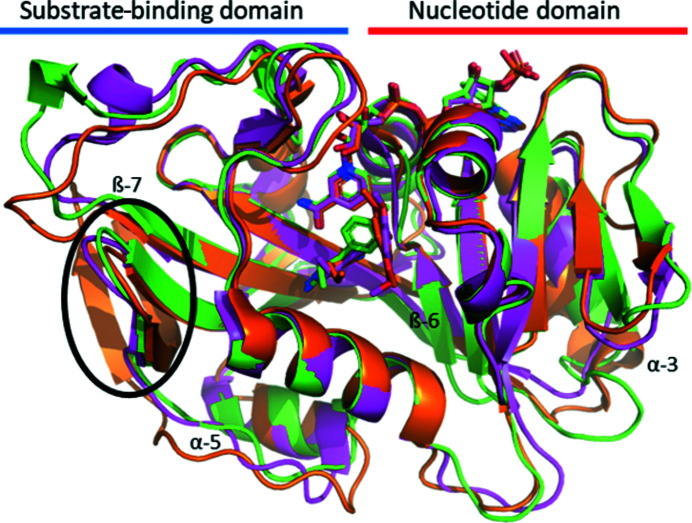
Superposition of CauDHFR (green; PDB entry 8a0z) with similar structures: CalDHFR (pink; PDB entry 3qlw) and CglDHFR (orange; PDB entry 3qlz). The two additional β-sheets in CglDHFR are circled in black.

As expected, two of the most closely related DHFR structures to CauDHFR are from the same genus: *C. albicans* (CalDHFR) and *C. glabrata* (CglDHFR). The sequence similarity of CauDHFR to CalDHFR and CglDHFR is about 42.8% and 44.2%, respectively (Supplementary Fig. S2). The crystal structures of CalDHFR and CglDHFR with a propargyl-based inhibitor have r.m.s.d.s of 0.50 and 0.65 Å, respectively, compared with the CauDHFR–NADPH–PYR complex (Fig. 2 ▸). The most conserved amino-acid sequence regions are observed to be surrounded by the substrate- and NADPH binding sites. In addition, while CauDHFR and CalDHFR have a central eight-stranded β-sheet, CglDHFR has two additional strands, giving a ten-stranded β-sheet, which contributes to the higher r.m.s.d. on superposition of these two structures, despite their greater sequence similarity. However, several loops in CauDHFR have significant conformational differences when compared with the DHFRs from the other two *Candida* species, particularly in the regions between α-helix 3 and β-sheet 6 and between β-sheet 7 and α-helix 5.

Overall, when the electrostatic surfaces are analysed there are some notable changes between the three *Candida* species. Asp160 in CauDHFR is substituted by Lys150 in CalDHFR, altering the charge of the region adjacent to α-helix 4 from strongly positive to negative (Supplementary Fig. S3*a* ). In CauDHFR, the region just after α-helix 1 is strongly positive, with multiple lysine residues: Lys5, Lys42 and Lys44 (Fig. 1 ▸ *f*). In contrast, in CalDHFR the same region contains a number of neutral residues, including multiple threonines, or negative residues, such as Asp170. On the other hand, the electrostatic surface of CauDHFR is similar to that of CglDHFR, particularly in the active site, with only slight alterations in the solvent-exposed regions (Supplementary Fig. S3*b* ).

#### 3.2.1. NADPH binding site

The NADPH binding site is located in a very positively charged cleft between α-helix 2 and α-helix 4, in which the cofactor is involved in an extensive number of hydrogen-bonding interactions (Supplementary Figs. S4*a* and S4*b* ). The diphosphate groups interact with both α-helix 2 and α-helix 4, specifically with Gly54, Thr57, Gly122, Ser123 and Gln124 (Supplementary Fig. S4). The adenine group interacts with Leu76, Ser77, Arg78 and Leu128 between α-helix 3 and α-helix 4, which is the most solvent-exposed moiety of NADPH (Supplementary Fig. S4*c* ). The ribose ring makes interactions with Ile19, Gly23 and Ser24 from the substrate-binding domain, which is the most neutral region of the cofactor-binding site. The nicotinamide moiety is buried in the active site of the enzyme, interacting with Ala11, Ile19, Leu25 and Tyr126, extending further towards the substrate-binding subdomain (Supplementary Fig. S4*d* ). This region is located between α-helix 2 and α-helix 4 and the pyrimidine rings of several clinically relevant antifolates form side–π interactions with the nicotinamide group of NADPH.

**Figure 3:**
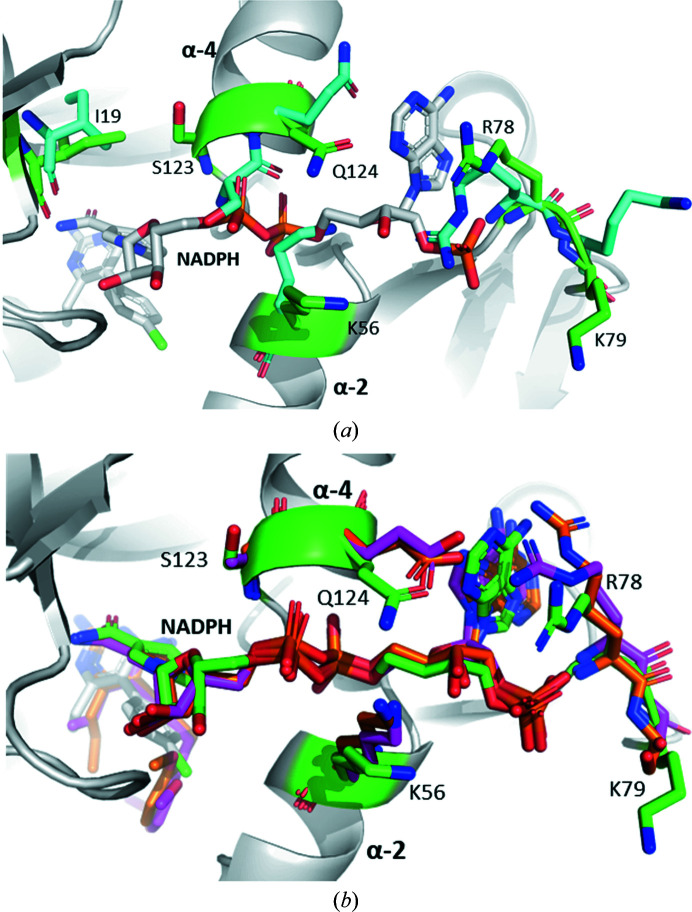
NADPH binding site and comparison to closely related enzymes. (*a*) Superimposed image of the NADPH binding site of the ternary complex of CauDHFR with NADPH and PYR (white; residues of interest are labelled in green; PDB entry 8a0z) and the CauDHFR apoenzyme (cyan, PDB entry 7zzx). (*b*) Superposition of the NADPH binding sites of the ternary complex of CauDHFR with residues of interest displayed (green; PDB entry 8a0z), CalDHFR (pink; PDB entry 3qlw) and CglDHFR (orange; PDB entry 3qlz).

Analysis of the NADPH binding sites of the four obtained structures shows that the holoenzyme and the CauDHFR–NADPH–PYR and CauDHFR–NADPH–CYG ternary complexes have the side chains of the NADPH binding site residues in similar conformations, whereas the apoenzyme has more significant differences. This could be caused by the conformational freedom imposed by the lack of cofactor. In the apoenzyme Lys56, Arg78 and Ser123 are in positions that occupy the NADPH binding site, whereas in the holoenzyme and the ternary complexes these are reorientated in order to interact with NADPH and avoid steric clashes (Fig. 3 ▸ *a*).

Comparing the structure of the CauDHFR–NADPH–PYR ternary complex with that of CalDHFR in complex with Asn22 (a propargyl-linked antifolate), the NADPH binding site is highly similar, with only small shifts of Lys56, Arg78 and Gln124 (Fig. 3 ▸ *b*) being observed. The Arg78 residue is significantly further away from the adenine moiety, at a distance of 4.2 Å compared with 3.3 Å for CalDHFR, and instead interacts with the phosphate moiety. The conformational shift of Gln124 in CauDHFR compared with Glu116 in CalDHFR is likely to lead to a minimal difference, as the carbonyl group remains in the same position at a distance of 3.3 Å from NADPH. Lys56 in both CauDHFR and CalDHFR is over 4 Å from NADPH and consequently does not disturb the interactions because of this conformational difference (Supplementary Fig. S5*b* ). The substitution of Ser123 in CauDHFR by Ala115 in CalDHFR and Gly124 in CglDHFR leads to the NADPH binding site being more hydrophobic in thes latter two species, although this does not seem to impact the NADPH–hydrogen interactions (Fig. 3 ▸ *b*).

Compared with CglDHFR, the NADPH binding site of CauDHFR is also largely similar, with some minor shifts of residues being observed. Arg78 in CglDHFR is highly similar to that in CalDHFR, being 3.3 Å from the adenine moiety compared with a distance of 4.2 Å in CauDHFR. Gln124 is in a similar conformation as in CalDHFR, with the carbonyl group staying in the same position and interacting with NADPH. The phosphate moiety and the nearby Arg55 residue are shifted by 1.0 Å, but the hydrogen-bonding interaction between them is maintained as both move by a similar distance (Supplementary Fig. S5*a* ).

### 3.3. Substrate-binding site of CauDHFR and comparison to other structures from *Candida* species

**Figure 4:**
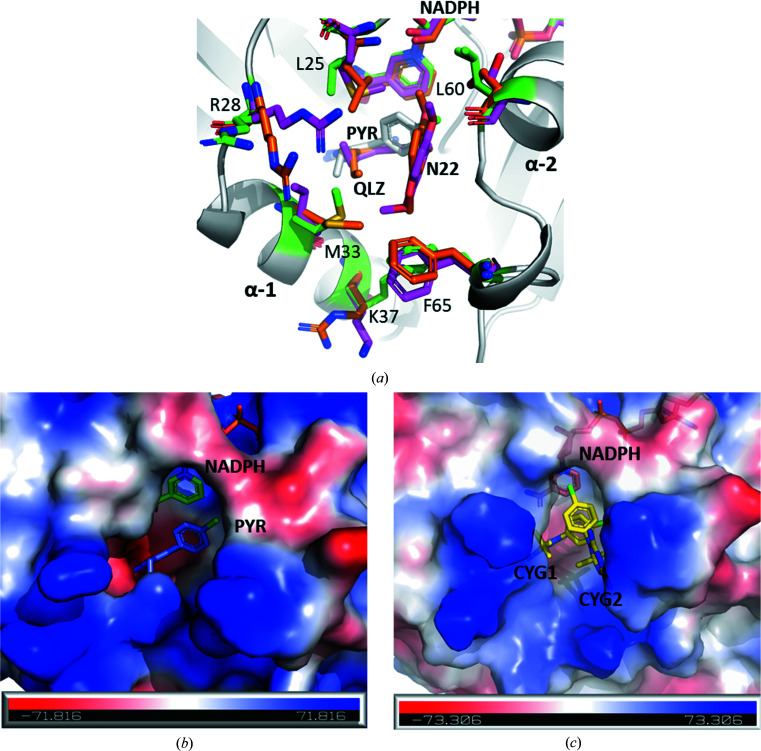
Substrate-binding site in CauDHFR. (*a*) Superposition of CauDHFR–NADPH–PYR (white; residues of interest are in green; PDB entry 8a0z), CalDHFR (pink; PDB entry 3qlw) complexed with the antifolate N22 and CglDHFR (orange; PDB entry 3qlz) complexed with the antifolate QLZ. (*b*) Electrostatic surface of the CauDHFR–NADPH–PYR ternary complex (NADPH in green and PYR in blue; PDB entry 8a0z). (*c*) Electrostatic surface of the CauDHFR–NADPH–CYG ternary complex (NADPH in pink and CYG in yellow; PDB entry 8crh).

The dihydrofolate (DHF) binding site is located in a pocket adjacent to the NADPH binding site (Fig. 4 ▸). The more external region of this site is positively charged, similar to those of other related species (Bhosle & Chandra, 2016 ▸), which is consistent with complementarity of charge with the substrate glutamate moieties. Deeper in the substrate pocket, the region surrounding the amide group of the nicotinamide moiety of NADPH is weakly negatively charged, particularly because of Glu32 and Thr141, and is complementary to the pteridine ring of DHF. In contrast, on the other side, which interacts with helix α2, the pocket is largely neutral and contains the hydrophobic residues Leu60 and Ile61, consistent with the PABA moiety of the substrate (Supplementary Fig. S6*a* ). DHF traditionally orientates itself in the active site such that the pteridine ring occupies a moderately negative region, whilst being close enough to engage in side–π interactions with the nicotinamide ring of NADPH. Classical antifolates also bind in this mode, with the pyrimidine or pteridine ring occupying the moderately negative region adjacent to NADPH and branching outwards, while larger antifolates such as MTX interact with the positive charge of the more external regions (Rajagopalan *et al.*, 2002 ▸).

The substrate-binding regions of apo and holo CauDHFR are largely similar, with the addition of NADPH not making a substantial difference to most residue orientations. The main difference observed is movement of Leu25, with a change of 5 Å between the isopropyl functional groups. This change in orientation is maintained in both ternary complexes. Minor movements are also seen in Met33, Thr57, Leu60 and Phe65 (Supplementary Fig. S6*b* ).

When comparing the DHF binding site in the three *Candida* species, notable differences are observed, including in the steric and electrostatic surface profiles (Fig. 4 ▸ *a*). Leu25 in CauDHFR is substituted by Met25 in CalDHFR, making this region more lipophilic and removing any possible interactions between the sulfur and substrate. In CglDHFR, this residue is not substituted but Leu25 is shifted deeper (by 0.9 Å) into the substrate pocket compared with in CauDHFR. On the other hand, Met33 in CglDHFR and CauDHFR is substituted by Ile33 in CalDHFR. This is a near-opposite change to the previously mentioned substitution, increasing the lipophilicity and removing possible sulfur–substrate interactions in this region. However, Met33 varies in position in CauDHFR and CglDHFR, with the residue pointing more into the active site in CauDHFR. Ser61 in CalDHFR and CglDHFR is substituted by Leu60 in CauDHFR, greatly reducing the hydrophilicity of the region and increasing the steric bulk, and also likely altering the positions of waters in the region. Arg28 is more wrapped around the active site in CalDHFR and CglDFR when compared with CauDHFR, where it is more exposed to the solvent. Both CalDHFR and CauDHFR have a Lys37 residue, while CglDHFR has an arginine at the same position. Since these residues have the same charge, a minor change is observed. Finally, the Phe65 residue varies slightly in all three *Candida* DHFR structures, with a different orientation in each structure. These small residue adjustments within the binding site have the potential to significantly alter the binding affinity of antifolates such as MTX.

### 3.4. Antifolate binding mode in CauDHFR

**Figure 5:**
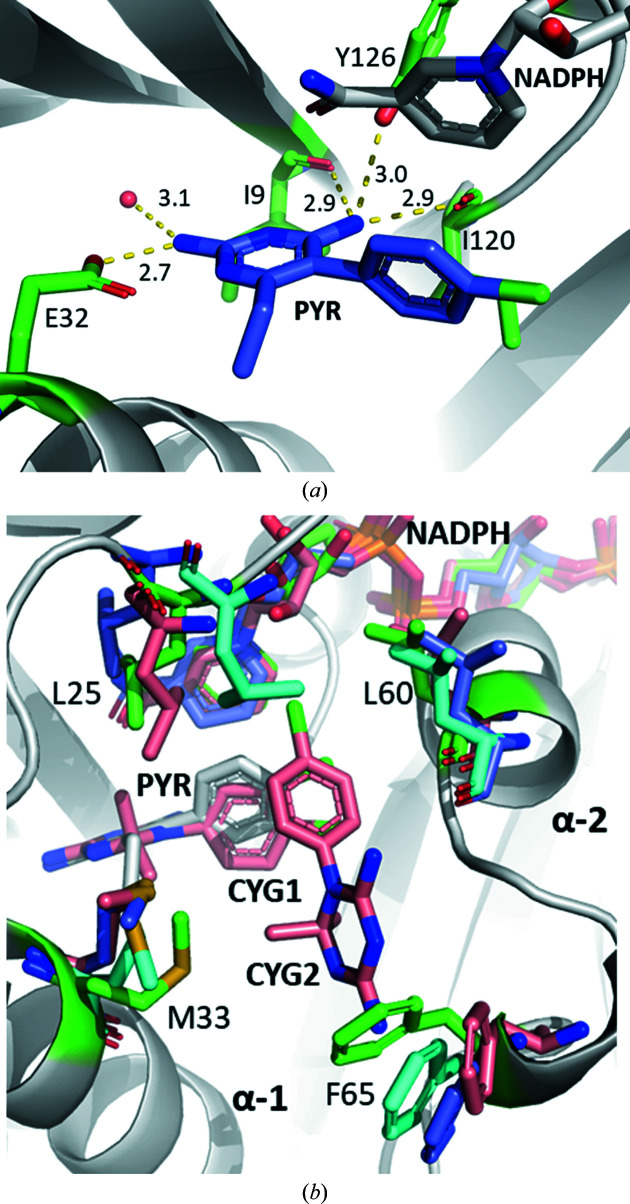
(*a*) Ternary complex of CauDHFR with NADPH and PYR, with the residues interacting with the amine groups of PYR highlighted (white; residues of interest in green and PYR in blue; PDB entry 8a0z). (*b*) Superposition of CauDHFR–NADPH–PYR (white; residues of interest in green; PDB entry 8a0z), the CauDHFR–NADPH–CYG ternary complex (pink; PDB entry 8crh), the CauDHFR apoenzyme (cyan; PDB entry 7zzx) and the CauDHFR holoenzyme (blue; PDB entry 8a0n).

In order to reveal the binding modes of PYR and CYG, we obtained the ternary complexes CauDHFR–NADPH–PYR and CauDHDFR–NADPH–CYG. PYR binds with the pyrim­idine ring in the negatively charged region adjacent to the nicotinamide moiety, with the amine deepest in the region and held in place via hydrogen-bond interactions with Glu32 and water held in a hydrophilic tunnel (Fig. 5 ▸ *a*). The other amine group interacts via hydrogen bonds to Tyr126 and the backbones of Ile9 and Ile120. Both rings interact with PYR via the nicotinamide region of NADPH via π–π and side–π inter­actions. The chlorine group of the chlorophenyl ring of PYR occupies the lipophilic region surrounding Leu60 and Ile61 (Fig. 4 ▸ *c*). Interestingly, two CYG molecules are clearly observed as electron density in the DHF binding site of CauDHFR (named CYG1 and CYG2 here; Supplementary Fig. S7). CYG1 binds in an almost identical manner to PYR, but a secondary CYG2 also binds within the active site (Fig. 4 ▸ *d*). For CYG2, the pyrimidine region interacts with the positively charged lip region, which is classically occupied by glutamate moieties, and the phenyl group occupies the apolar region that is classically occupied by the PABA moiety, stacked above the first CYG compound.

Comparing the structures of apo and holo CauDHFR and the complexes with PYR or CYG, there are slight conformational differences in the residues within the DHF binding site (Fig. 5 ▸ *b*). Overall, we observe that several residues move into the active site, closing it. In this regard, Leu25 moves about 1.8 Å into the active site when PYR is bound compared with the holoenzyme and apoenzyme structures. Ile61 in CauDHFR–NADPH–PYR also shifts 1.2 and 1.5 Å further into the active site compared with the apoenzyme and holoenzyme, respectively. Met33 follows this trend, with differences of 3.6 and 3.3 Å for the apoenyzme and holo­enzyme, respectively. Phe65 is also shifted significantly into the active site in the presence of PYR, moving 2.2 and 3.3 Å compared with the apoenyzme and holoenzyme, respectively. The ternary complex with CYG only has slight conformational differences compared with the ternary complex with PYR, except for Phe65, which moves by 2.6 Å. This is most likely to be due to the steric clash that would arise in the ternary complex with CYG if it was in the same position as in the ternary complex with PYR. The Leu60 residue is shifted towards the NADPH molecule in both ternary complexes, being shifted 1.4 and 2.1 Å from the apoenzyme and holo­enzyme, respectively, with respect to CauDHFR–NADPH–PYR and 1.1 and 1.6 Å, respectively, with respect to CauDHFR–NADPH–CYG.

### 3.5. Comparison of the CauDHFR substrate-binding site with that of *Homo sapiens* DHFR (HsDHFR)

**Figure 6:**
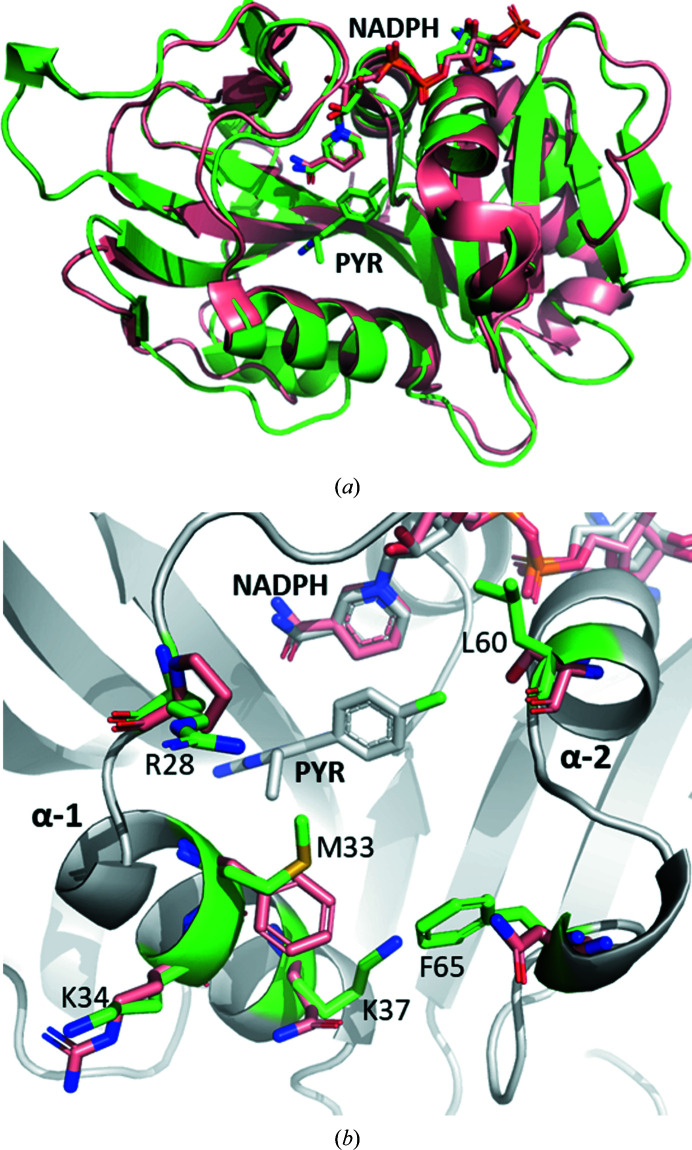
Active-site superposition of CauDHFR and HsDHFR. (*a*) Superposition of *C. auris* DHFR (green; PDB entry 8a0z) and *H. sapiens* DHFR (salmon; PDB entry 4m6j). (*b*) Superposition of CauDHFR–NADPH–PYR (white; residues of interest in green; PDB entry 8a0z) and HsDHFR (salmon; PDB entry 4m6j).

For a drug-discovery campaign, selectivity for CauDHFR over the human analogue is essential. The r.m.s.d. between CauDHFR and HsDHFR is 0.84 Å, which is slightly greater than that between CauDHFR and its closest analogues. The sequence similarity is also lower at only 35%, and consequently there are many residue substitutions that could guide the design of specific inhibitors (Fig. 6 ▸). The most significant substitutions include the substitution of Met33 in CauDHFR by Phe31 in HsDHFR, which substantially increases the steric bulk and the lipophilicity of the region. Phe65 at the entrance to the active site in CauDHFR is swapped for Asp64, which corresponds to a large increase in hydrophilicity and a reduction in steric bulk compared with the phenyl ring. As in the other *Candida* species, Leu60 in CauDHFR is exchanged for Ser59, a more hydrophilic residue that is capable of hydrogen bonding. Lys37 of CauDHFR is substituted by Gln35, maintaining the hydrophilicity of the region, but reducing the positive charge of the active-site lip region. Lys34 in CauDHFR is changed to Arg32 in HsDHFR, which is a minor alteration since both residues are positively charged. Arg28 in CauDHFR is altered to Pro26, a substantial steric alteration that reduces the positive electrostatics in the region. The electrostatic surface of the active site for HsDHFR remains largely similar, but the entrance to the active site is reduced in positive charge, and the residue shifts also make this region more exposed to solvent. Calculating the pocket of the active site using *CASTp* (Binkowski *et al.*, 2003 ▸), these residue substitutions cause an increase in the volume from 598 to 654 Å^3^ for CauDHFR and HsDHFR, respectively.

### 3.6. Biochemical characterization of CauDHFR

For further characterization, a collection of biochemical assays were performed to assess the inhibitory effect of common antifolates. Initial *in vitro* studies of the thermal shift showed MTX to have the highest shift of +17°C, with PYR having a lower shift of +9.5°C, CYG having a shift of +9.0°C and TMP having the lowest shift of +6.0°C.

CauDHFR activity could also be monitored using 340 nm ultraviolet light, which shows the consumption of NADPH as it is converted to NADP^+^, enabling the reduction of dihydrofolate to tetrahydrofolate. NADPH oxidation assays to obtain IC_50_ mainly reproduced the trend from the thermal shift, with MTX being the most active at 31 n*M*, followed by PYR and TMP. The exception to this trend was CYG, with a significantly lower IC_50_ than would be expected from the thermal shift at 401 µ*M*. This is a surprising result due to its largely similar crystal structure when compared with the CauDHFR–NADPH–PYR complex. This change may be due to the lack of aromaticity in CYG or to the dimethyl group leading to a higher energy water conformation within the active site (Supplementary Fig. S8).

Studies were then performed against *Candida* spp. yeasts, with the addition of two more antifolates: diaveridine (DIA) and pemetrexed (PMX) (Supplementary Table S1). The inhibitory effect of these inhibitors on the fungal growth of *C. albicans*, *C. glabrata* and *C. auris* was tested using the broth microdilution assay. Among the DHFR inhibitors, PYR was the most active in inhibiting *Candida* spp., including *C. auris* (125 µg ml^−1^), with all other antifolates tested having an MIC of greater than 500 µg ml^−1^ against *C. auris* (Table 2 ▸). Among PYR, MTX and CYG, CYG was the least active against *Candida* spp. (Supplementary Table S1), including *C. auris*, supporting the lack of affinity seen in the IC_50_ assay (Table 2 ▸). Interestingly, PYR was more active against *C. glabata* and *C. albicans*, with an MIC of 62.5 µg ml^−1^, showing additional difficulties in inhibiting *C. auris* even over other *Candida* species.

## 4. Conclusion

In summary, we obtained crystal structures of CauDHFR as an apoenzyme and a holoenzyme and in ternary complexes with two antifolates, PYR and CYG, at 2.4, 1.4, 1.7 and 1.3 Å resolution, respectively. The structures obtained are similar to those from related species such as *C. albicans* and *C. glabrata*. However, the structures of these enzymes show some important differences within the different complexes and in the cofactor- and substrate-binding sites. Compared with human DHFR, there are a wide array of residue substitutions in the active site, leading to a different spatial and electrostatic environment that could be explored in the design of specific inhibitors of *C. auris*. In addition to this, biochemical assays were performed, assessing the efficacy of common antifolates against CauDHFR and *C. auris*. Taking these structural and biochemical data together, modification of current antifolates and structure-based expansions might be attempted, aiming towards the elaboration of compounds with high affinity that are selective against CauDHFR.

## Supplementary Material

PDB reference: *Candida auris* dihydrofolate reductase, apo form, 7zzx

PDB reference: complex with NADPH, 8a0n

PDB reference: complex with NADPH and pyrimethamine, 8a0z

PDB reference: complex with NADPH and cycloguanil, 8crh

Supplementary Figures and Table. DOI: 10.1107/S2059798323004709/ji5031sup1.pdf

## Figures and Tables

**Table 1:** Crystallographic statistics for X-ray data processing, structure refinement and stereochemistry of CauDHFR structures

	Apo CauDHFR	CauDHFR–NADPH	CauDHFR–NADPH–PYR	CauDHFR–NADPH–CYG
PDB code	7zzx	8a0n	8a0z	8crh
Conditions	1.6 *M* sodium citrate pH 6.3	0.2 *M* NaNO_3_, 20% PEG 3350	0.2 *M* NaNO_3_, 20% PEG 3350	0.2 *M* Li_2_SO_4_, 0.1 *M* Tris, 27.5% PEG 3350 pH 8.5
Temperature (K)	100	100	100	100
Wavelength (Å)	1.0000	1.0000	1.0000	1.0000
Resolution range (Å)	35.14–2.40 (2.49–2.40)	38.26–1.40 (1.45–1.40)	33.27–1.70 (1.76–1.70)	42.36–1.30 (1.346–1.30)
Space group	*P*3_1_21	*P*2_1_2_1_2_1_	*P*1	*P*2_1_2_1_2_1_
*a*, *b*, *c* (Å)	146.3, 146.3, 31.5	43.0, 63.3, 76.5	41.2, 45.4, 54.1	43.8, 63.6, 167.6
α, β, γ (°)	90, 90, 120	90, 90, 90	105.3, 93.3, 90.2	90, 90, 90
Total reflections	300978 (25112)	530806 (34787)	121461 (4544)	1304270 (45214)
Unique reflections	15279 (1503)	41455 (3813)	34617 (1481)	112746 (9094)
Multiplicity	19.7 (16.7)	12.8 (9.1)	3.5 (3.1)	11.6 (4.9)
Completeness (%)	99.80 (99.40)	98.61 (89.53)	83.19 (35.58)	96.97 (79.51)
〈*I*/σ(*I*)〉	12.84 (0.81)	18.19 (1.07)	22.29 (3.40)	19.59 (0.80)
Wilson *B* factor (Å^2^)	55.45	16.77	17.99	16.11
*R*_merge_	0.1680 (2.036)	0.0690 (0.815)	0.0522 (0.234)	0.0480 (0.409)
*R*_meas_	0.1722 (2.101)	0.0715 (0.863)	0.0613 (0.281)	0.0501 (0.458)
*R*_p.i.m._	0.0387 (0.514)	0.0197 (0.274)	0.0319 (0.153)	0.0140 (0.200)
CC_1/2_	0.999 (0.760)	1 (0.844)	0.998 (0.940)	0.999 (0.907)
CC*	1 (0.929)	1 (0.957)	0.999 (0.984)	1 (0.975)
Reflections used in refinement	15262 (1503)	41290 (3676)	34572 (1479)	112511 (9094)
Reflections used for *R*_free_	1525 (150)	1986 (174)	1995 (90)	1995 (160)
*R*_work_	0.205 (0.291)	0.176 (0.259)	0.190 (0.235)	0.170 (0.320)
*R*_free_	0.230 (0.323)	0.200 (0.266)	0.223 (0.261)	0.183 (0.336)
CC(work)	0.961 (0.764)	0.966 (0.891)	0.947 (0.916)	0.969 (0.631)
CC(free)	0.935 (0.780)	0.954 (0.870)	0.936 (0.918)	0.971 (0.650)
No. of non-H atoms				
Total	1677	2201	3827	4198
Macromolecules	1636	1750	3303	3454
Ligands	0	60	168	121
Solvent	41	391	356	623
Protein residues	202	213	408	409
R.m.s.d., bond lengths	0.010	0.008	0.009	0.008
R.m.s.d., angles	1.28	1.33	1.43	1.32
Ramachandran favoured (%)	91.50	98.10	98.50	98.52
Ramachandran allowed (%)	6.50	1.90	1.50	1.23
Ramachandran outliers (%)	2.00	0.00	0.00	0.25
Rotamer outliers (%)	0.56	0.00	0.00	0.00
Clashscore	13.80	7.73	15.45	4.53
Average *B* factor (Å^2^)				
Overall	69.34	22.52	22.12	24.30
Macromolecules	69.65	20.16	21.36	22.37
Ligands	0	18.80	21.68	25.90
Solvent	57.02	33.65	29.43	34.68
No. of TLS groups	1	1	1	1

**Table 2:** Biochemical data for CauDHFR in the presence of four common antifolates: methotrexate (MTX), pyrimethamine (PYR), trimethoprim (TMP) and cycloguanil (CYG)

	MTX	PYR	TMP	CYG
Thermal shift (°C)	+17.0	+9.5	+6.0	+9.0
IC_50_ (µ*M*)	0.031	5.2	73.6	401
MIC (µg ml^−1^)	>500	125	>500	>500
