# Supplementary material for: Crystal structure of dihydrofolate reductase from the emerging pathogenic fungus Candida auris
Source: Acta Crystallogr D Struct Biol. 2023 Jul 10;79(Pt 8):735–45. doi: 10.1107/S2059798323004709 (PMC10394672; doi:10.1107/S2059798323004709)
Supplement: Supplementary file 1 [file d-79-00735-sup1.pdf]

STRUCTURAL BIOLOGY

Volume 79 (2023)

Supporting information for article:

Crystal structure of dihydrofolate reductase from the emerging pathogenic fungus *Candida auris*

Tim Kirkman, Alice Sketcher, Vinicius de Morais Barroso, Kelly Ishida, Manuela Tosin and Marcio Vinicius Bertacine Dias

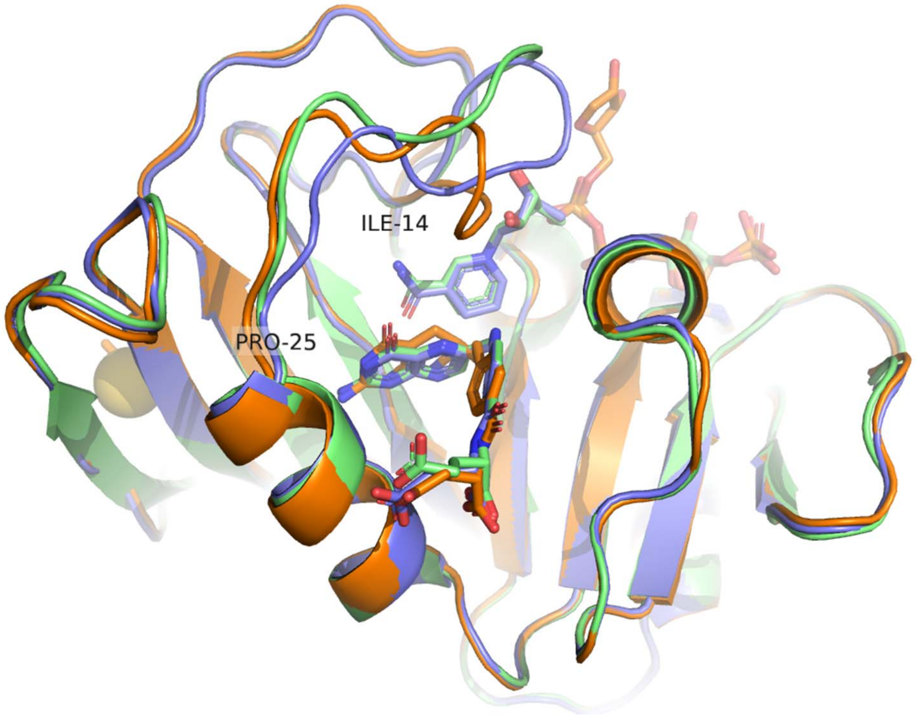

Figure S1 Superposition of EcDHFR in the open conformation (green, PDB: 1RA2), closed conformation (blue, PDB: 1RX2), and occluded conformation (orange, PDB: 1RX6).

```
CauDHFR      MSTRPKISLIVAALQPSMGIGAKGSLPWRLKNEMKYFKDVTSKAK-DGHINAVVMGRKTW    59
CglDHFR      -MSKVPVVGIVAALLPEMGIGFQGNLPWRLAKEMKYFREVTTLTNDNSKQNVVIMGRKTW    59
CalDHFR      -MSKPNVAIIVAALKPALGIGYKGKMPWRLRKEIRYFKDVTTRTTKPNTRNAVIMGRKTW    59
               ::  :  ***** * :*** :*.:**** :*::**::**: :.  .  *.*:******

CauDHFR      ELIPERFRPLAGRLNVILSRKNDDLI--DSNGVYHFSSFDSVMKHLEKDSFRFKDMPLDK    117
CglDHFR      ESIPQKFRPLPKRINVVVSRSFDGELRKVEDGIYHSNSLRNCLTALQSS--LANENKIER    117
CalDHFR      ESIPQKFRPLPDRLNIILSRSYENEI--IDDNIIHASSIESSLNLV---------SDVER    108
             * **::****  *:*:::**. :. :   .:.: * .*: . :. :           :::

CauDHFR      IFIIGGSQIYNLLILDSRVDNLLVTQVHFVGEDADKPQMDTFLDWDLSK-WKRLEHDK--    174
CglDHFR      IYIIGGGEIYRQSMD--LADHWLITKIMPL-PETTIPQMDTFLQKQELEQRFYDNSDKLV    174
CalDHFR      VFIIGGAEIYNELINNSLVSHLLITEIEHP--SPESIEMDTFLKFPLES-WTKQPKSE--    163
             ::****.:**.  :    ..: *:*::     .    :*****.    .       .:

CauDHFR      --------------LEQYVGLDVPRGLNEEGSYNYEYTMWEKAQ   204
CglDHFR      DFLPSSIQLEGRLTSQEWNGELVKGLPVQEKGYQFYFTLYTKK-   217
CalDHFR      --------------LQKFVGDTVLEDDIKEGDFTYNYTLWTRK-   192
                            ::: *  *     :* .: : :*:: :
```

Figure S2 Sequence alignment data of *Cau*DHFR, *Cgl*DHFR, and *Cal*DHFR by Clustal Omega (residues are coloured according to their physiochemical properties: Magenta - basic, Blue - acidic, Red - small + hydrophobic, Green - Hydroxyl + sulfhydryl + amine + glycine).

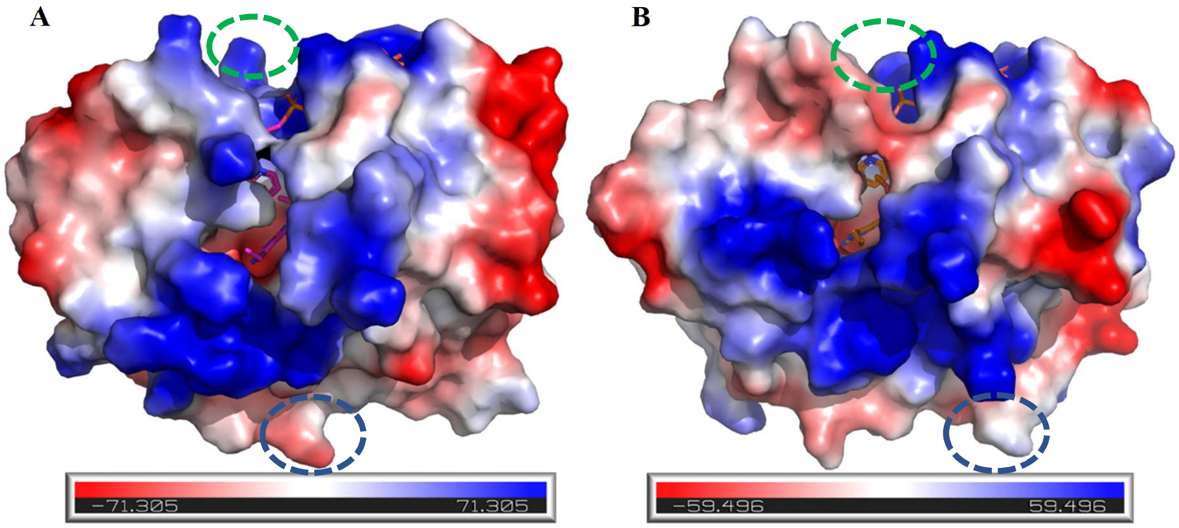

Figure S3 (A) Electrostatic surface of CalDHFR in complex with a propargyl-linked antifolate with regions if interest circled (PDB: 3QLW). (B) Electrostatic surface of CglDHFR in complexed with a propargyl-linked antifolate with regions of interest circled (PDB: 3QLZ). Regions circled in identical position to that for Figure 1F to highlight differences.

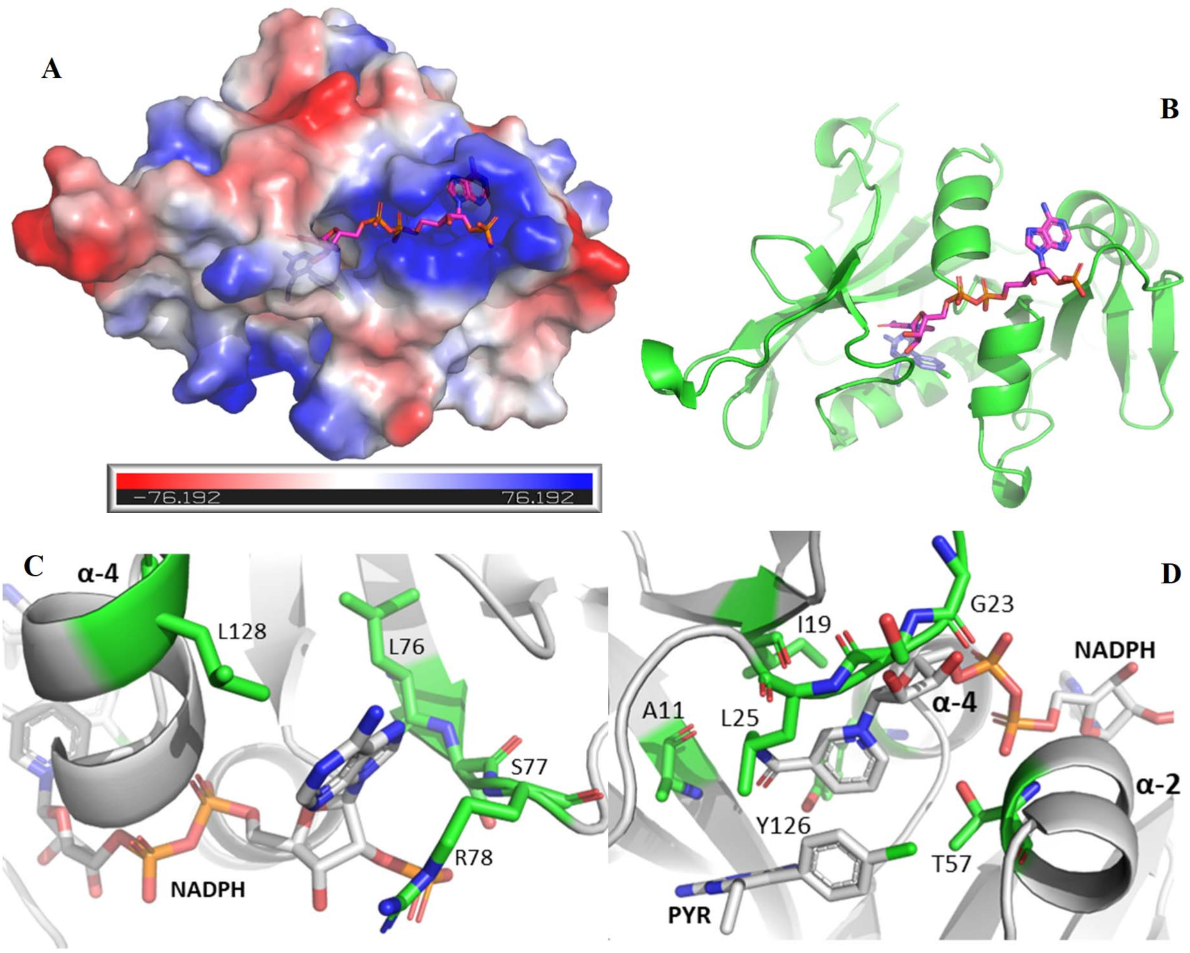

Figure S4 (A) Electrostatic surface of CauDHFR:NADPH:PYR ternary complex orientated to show NADPH (NADPH pink, PDB: 8A0Z). (B) Cartoon image of CauDHFR:NADPH:PYR ternary complex (green, NADPH pink, PDB: 8A0Z). (C) CauDHFR:NADPH:PYR ternary complex focused on adenine moiety of NADPH with nearby residues labelled. (D) CauDHFR:NADPH:PYR ternary complex focused on nicotinamide moiety of NADPH with nearby residues labelled.

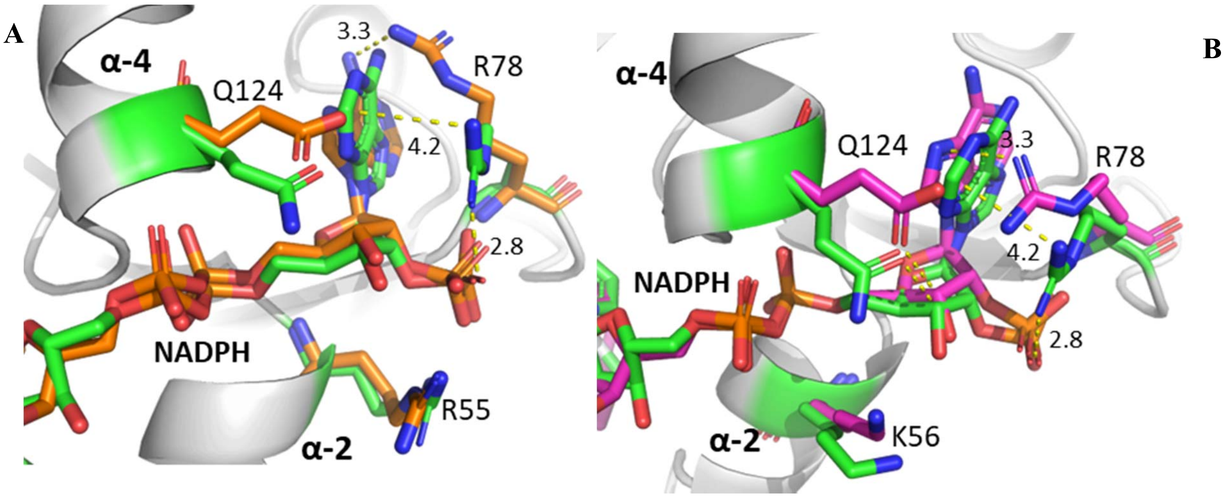

Figure S5 (A) Superposition of CauDHFR:NADPH:PYR (white, residues of interest green) and residues of CglDHFR holoenzyme (PDB: 3QLZ, orange) focused around the adenine moiety of NADPH. (B) Superposition of CauDHFR:NADPH:PYR (white, residues of interest green) and residues of CalDHFR holoenzyme (PDB: 3QLW, pink) focused around the adenine moiety of NADPH.

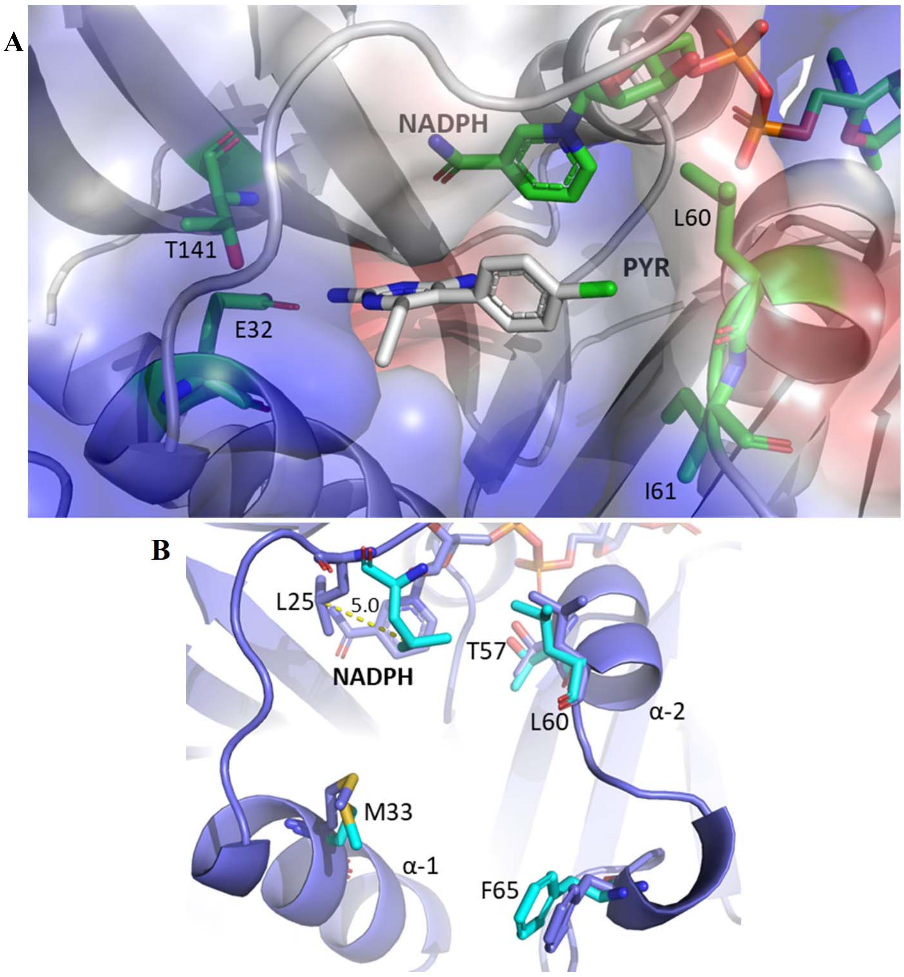

Figure S6 (A) Residues denoting electronegativity in substrate binding site adjacent to NADPH for CauDHFR:NADPH:PYR. (B) Superposition of CauDHFR holoenzyme (blue) and residues of CauDHFR apoenzyme (cyan) with the distance between the L25 residues shown.

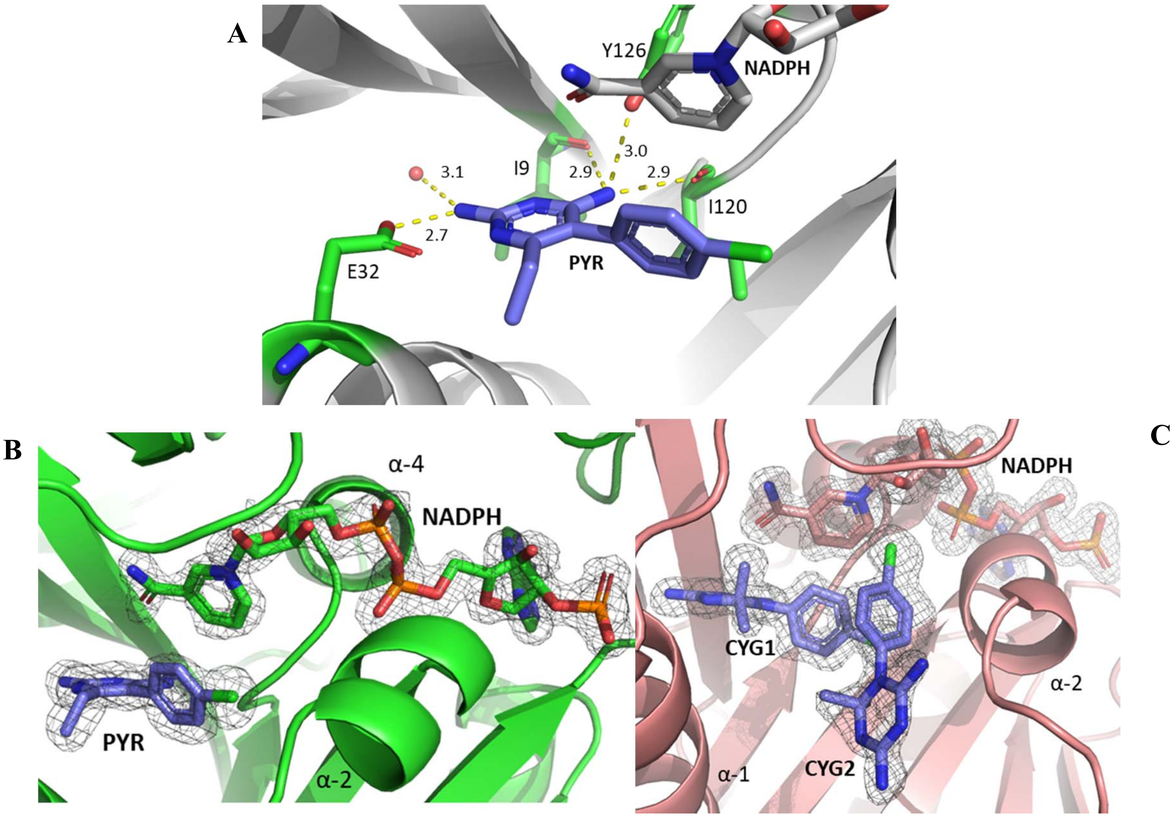

Figure S7 (A) CauDHFR ternary complex with NADPH and PYR, with residues interacting with amine groups of PYR highlighted (white, residues green, PYR blue, PDB: 8A0Z). (B) CauDHFR ternary complex with NADPH and PYR, with 2Fo-Fc electron density map of PYR and NADPH (green, PDB: 8A0Z) at 1σ. (C) CauDHFR ternary complex with NADPH and two CYG molecules, with 2Fo-Fc electron density map of CYGs and NADPH Å (rose, PDB: 8CRH) at 1σ.

Table S1 Minimum inhibitory concentration (MIC) of DHFR inhibitors against *Candida* spp. growth.

Strains	MIC (μg/ml)					
	trimethoprim	methotrexate	pemetrexed	diaveridine	pyrimethamine	Cycloguanil
C. albicans	500	250	500	>500	62.5	500
C. glabrata	500	>500	500	500	62.5	500
C. auris	>500	>500	>500	>500	125	>500

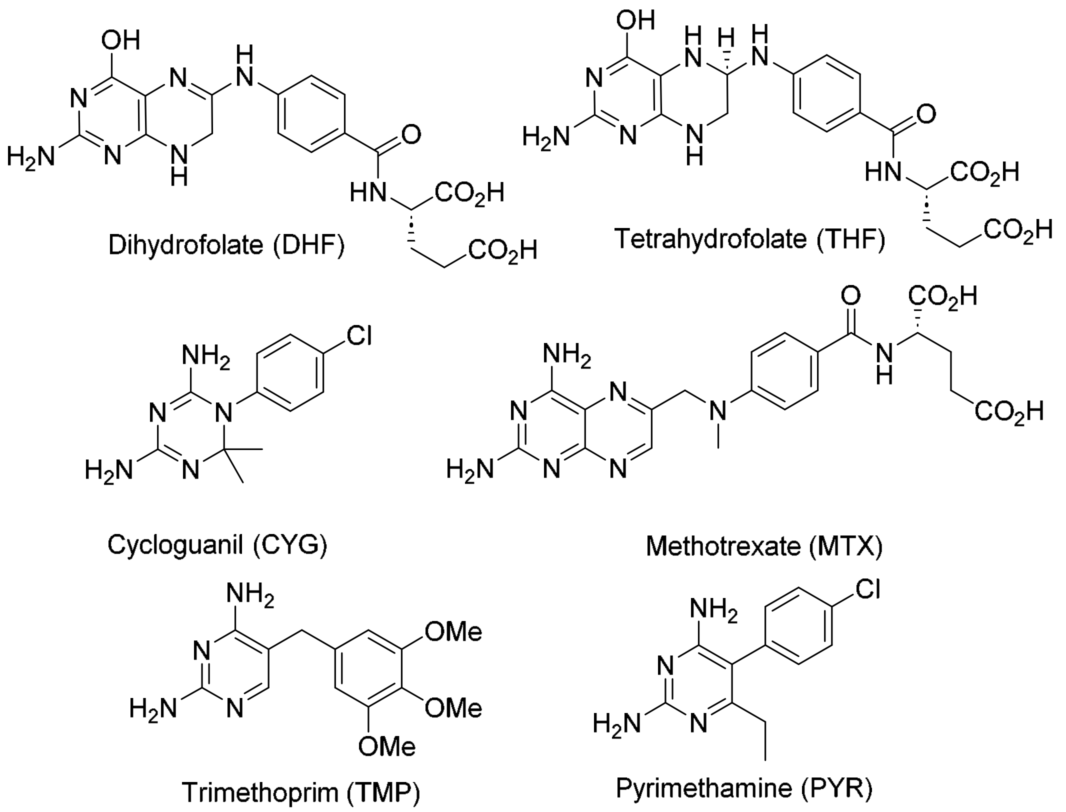

Figure S8 Chemical structures of compounds discussed.
